# Forecasting COVID-19 spreading through an ensemble of classical and machine learning models: Spain’s case study

**DOI:** 10.1038/s41598-023-33795-8

**Published:** 2023-04-25

**Authors:** Ignacio Heredia Cacha, Judith Sáinz-Pardo Díaz, María Castrillo, Álvaro López García

**Affiliations:** Instituto de Física de Cantabria (IFCA), CSIC-UC, Avda. los Castros s/n., 39005 Santander, Spain

**Keywords:** Computational science, Scientific data, Statistics, Mathematics and computing, Information technology

## Abstract

In this work the applicability of an ensemble of population and machine learning models to predict the evolution of the COVID-19 pandemic in Spain is evaluated, relying solely on public datasets. Firstly, using only incidence data, we trained machine learning models and adjusted classical ODE-based population models, especially suited to capture long term trends. As a novel approach, we then made an ensemble of these two families of models in order to obtain a more robust and accurate prediction. We then proceed to improve machine learning models by adding more input features: vaccination, human mobility and weather conditions. However, these improvements did not translate to the overall ensemble, as the different model families had also different prediction patterns. Additionally, machine learning models degraded when new COVID variants appeared after training. We finally used Shapley Additive Explanation values to discern the relative importance of the different input features for the machine learning models’ predictions. The conclusion of this work is that the ensemble of machine learning models and population models can be a promising alternative to SEIR-like compartmental models, especially given that the former do not need data from recovered patients, which are hard to collect and generally unavailable.

## Introduction

After the surge of cases of the new Coronavirus Disease 2019 (COVID-19), caused by the SARS-COV-2 virus, several measures were imposed to slow down the spread of the disease in every region in Spain by the second week of March 2020. Over the time, these measures have included hard lock-downs, restrictions on people mobility, limitations of the number of people in public places and the usage of protection gear (masks or gloves), among others.

The application of those measures has not been consistent between countries nor between Spain regions. This makes it hard to reliably assess the impact of the individual restrictions to avoid the spreading^1,2^. Human mobility and its direct impact on the spread of infectious diseases (including COVID-19) has been profusely studied, and restricting or limiting the mobility from infected areas is one of the first measures being adopted by authorities in order to prevent an epidemic spread, with different results^2–8^. In addition, weather conditions have an influence on the evolution of the pandemic, as it is known that other respiratory viruses survive less in humid climates and with low temperatures^9^. Some studies already evaluated the influence of climate on COVID-19 cases, for example^10^, where it is concluded that climatic factors play an important role in the pandemic, and^11^, where it is also concluded that climate is a relevant factor in determining the incidence rate of COVID-19 pandemic cases (in the first citation this is concluded for a tropical country and in the second one for the case of India).

In this context, the approach that we propose in this work is to predict the spread of COVID-19 combining both machine learning (ML) and classical population models, using exclusively publicly available data of incidence, mobility, vaccination and weather. Having a reliable forecast enables us to assess the influence of these factors on the spreading rate, thus allowing decision makers to design more effective policies.

The motivation for using these two types of models lies in the fact that, from our experience, while ML models in the vast majority of cases overestimate the number of daily cases, population models generally seem to predict fewer cases than the actual ones. To make the most of both model families, we aggregated their predictions using ensemble learning. In ensemble learning all the individual predictions are combined to generate a meta-prediction and the ensemble usually outperforms any of its individual model members^12,13^.

The contributions made in the present work can be summarized in two essential points:

- Classical and ML models are combined and their optimal temporal range of applicability is studied. We are currently not aware of any work including an ensemble of both ML and population models (ODE based) for epidemiological predictions.
- As classical models, less explored population growth models are used. Contrary to compartmental epidemiological models, these models can be used even when the data of recovered population are not available. This is a crucial advantage because recovered patient data are usually hard to collect, and in fact not available anymore for Spain since 17 May 2020 (see dataset in^14^). It should be noted nevertheless that some regions do provide these data on recoveries and/or active cases, and there are some very successful works in the development of this type of compartmental models^15^.

The paper is structured as follows: section “Related work” contains the related work relevant to this publication; section “Data” outlines the datasets considered for our work, as well as the pre-processing that we have performed to them; in section “Methods” we present the ensemble of models being used to predict the evolution of the epidemic spread in Spain; section “Results and discussion” describes our main findings and results; section “Conclusions” contains the main conclusions which emerge from the analysis of results and the last one (section “Challenges and future directions”) outlines the future work which arises from this research.

## Related work

Much effort has been done to try to predict the COVID-19 spreading, and therefore to be able to design better and more reliable control measures^16^. Many of the most solid work comes from classical compartmental epidemiological models like SEIR, where population is divided in different compartments (**S**usceptible, **E**xposed, **I**nfected, **R**ecovered). Many SEIR models have been extended to account for additional factors like confinements^17^, population migrations^18^, types of social interactions^19^ or the survival of the pathogen in the environment^20^. In particular,^15^ predicts required beds at Intensive Care Units by adding 4 additional compartments to those of the SEIR model: Fatality cases, Asymptomatics, Hospitalized and Super-spreaders.

In the present study, instead of compartmental models we chose to use population models, for which we only need the data of the daily cases. Several works already include the use of this type of models for the COVID-19 case studies, such as^21^, where the use of Gompertz curves and logistic regression is proposed, or^22^, where the Von Bertalanffy growth function (VBGF) is used to forecast the trend of COVID-19 outbreak. Additionally,^23^ compares the use of artificial neural networks and the Gompertz model to predict the dynamics of COVID-19 deaths in Mexico. However, our approach does not compare the performance of both kind of models (ML and population models), instead it combines them to try to obtain more accurate and robust predictions.

In recent years, ML has emerged as a strong competitor to classical mechanistic models. In the context of the spread of COVID-19 during the early phases of the outbreak, the focus was on trying to predict the evolution of the time series of pandemic numbers^24,25^, with disparate prediction quality and uncertainties. ML has been used both as a standalone model^26^ or as a top layer over classical epidemiological models^27^. ML models have been used to exploit different big data sources^28,29^ or incorporating heterogeneous features^30^. Also, several general evaluations of the applicability of these models exist^31–34^. Applications of deep learning techniques arise beyond the classically expected for dealing with COVID-19 (e.g. epidemiology), such as Natural Language Processing (NLP) or computer vision through the use of deep learning techniques, are also as reported in^35^.

Regarding the model ensemble, work has been developed both in the USA^36^ and EU^37^ to consolidate all these different models by deploying portals that ensemble the predictions. ML techniques have also been used to help improving classical epidemiological models^38^.

Despite everyone best efforts, sensible work has carefully warned against the possibility of meaningfully predicting the evolution for temporal horizons over a week^39^, just as is the case for the weather forecasts. For this reason, we do our best all over this paper to point out the limitations of our data (as presented at the end of the next section) and models so that we do not add more fuel to the hype wagon.

## Data

In the spirit of Open Science, the present work exclusively relies on open-access public data. The intention is, one the hand, to contribute to the rigorous assessment of the models before they can be adopted by policy makers, and on the other hand to encourage the release of comprehensive and quality open datasets by public administrations, not limited to the COVID-19 pandemic data.

Our dataset is composed of COVID-19 cases data, COVID-19 vaccination data, human population mobility data and weather observations, and is constructed as explained in what follows.

The spatial basic units of the present work are the whole country (Spain), and the autonomous community (Spain is composed of 17 autonomous communities and 2 autonomous cities). Therefore, the final objective is to predict the number of daily cases per day for Spain as a whole and for each autonomous community. Due to their particular geographical situation and demographics, the pandemic outbreak in the two autonomous cities of Ceuta and Melilla had a different behaviour and they have not been analyzed individually in this study. However, we have considered the daily cases reported by these autonomous cities in the total number of daily cases in Spain. Furthermore, in the case of mobility and temperature, these data are different if the analysis is carried out for the whole of Spain, or if it is done by autonomous community.

**Figure 1:**
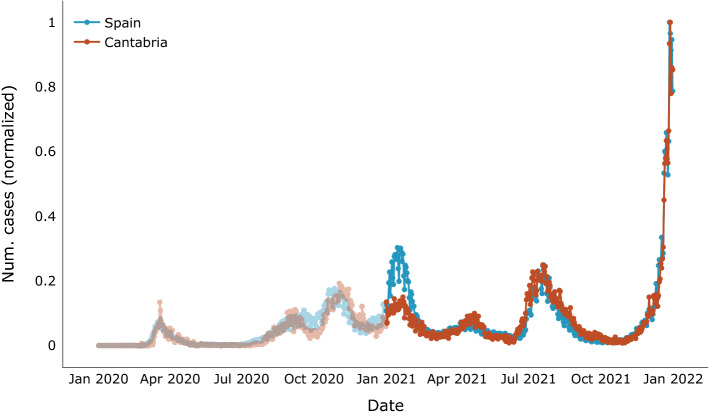
Daily COVID-19 confirmed cases (normalized) in Spain and in Cantabria autonomous community. Transparency is added to data outside our considered time range (data before 2021).

The dataset time range goes from January 1st, 2021 to December 31st, 2021. For consistency, we do not include data before that date because vaccination in Spain started on December 27st, 2020. Also, note that after November 2021, the daily cases exploded due to Omicron variant (cf. Fig. 1), so the forecasts will be presumably worse in that month.

In the case of the ML models, these data were split into training, validation and test sets. Specifically, the days to be predicted in test were, from October 2nd, 2021 (so the date on which the prediction would be made is October 1st), until December 31st. The 30 days prior to these dates correspond to the validation set, and the rest to the training set. Note that forecasts are made for 14 days. In the case of the population models, we considered the same test set, and as training the 30 days prior to the 14 days to be predicted (more details in section “Population models”).

### Daily COVID-19 cases data

Concerning the data on daily cases confirmed by COVID-19, we used the data collected by the Carlos III Health Institute —in Spanish *Instituto de Salud Carlos III (ISCIII)*—which is a Spanish autonomous public organization currently dependent on the Ministry of Science and Innovation—in Spanish *Ministerio de Ciencia e Innovación (MICINN)*—. The data source is available in^40^.

The dataset classifies new cases according to the test technique used to detect them (PCR, antibody, antigen, unknown) and the autonomous community of residence. For this study, we used the total number of new cases across all techniques.

Figure 1 shows the evolution of daily COVID-19 cases (normalized) throughout 2021 for Spain, and for the autonomous community of Cantabria as an example. It reveals that the evolution of the trend for Cantabria is analogous to that of the country as a whole.

**Figure 2:**
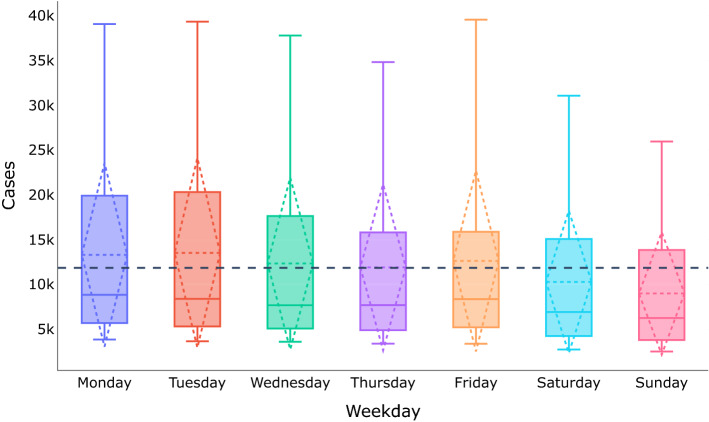
Statistics on the number of cases depending on the day of the week (ML train set). The dotted black line shows the mean of the daily cases in the study period, and in each boxplot the mean and standard deviation are also shown as dashed lines

Figure 2 shows the number of diagnosed cases according to the day of the week when they were recorded. As expected, a weekly pattern is perceived, with a lower number of cases recorded on the weekends. However, after performing some preliminary tests as they are explained later, finally the day of the week was not included as an input variable in the models.

### Vaccination data

Vaccination against COVID-19 has shown as key to protect the most vulnerable groups, reducing the severity and mortality of the disease. The vaccination process in Spain began on December 27th, 2020, prioritizing its inoculation to people living in elderly residences and other dependency centers, health personnel and first-line healthcare partners, and people with a high degree of dependency not institutionalized. The vaccination strategy continued with the most vulnerable people following an age criterion, in a descending order. By June 2021, the vaccine was widely available, and the process continued again in descending order of age, reaching those over 12 years of age. Thus, by October 14th, 87.9$\%$ of the target population (i.e. those over 12 years old) had received the full vaccination schedule^41^.

As of December 15th, 2021, 4 vaccines were authorized for administration by the European Medicines Agency (EMA)^41^ (cf. Table 1).

**Table 1:** Vaccines authorized by the EMA.

Abbrv.	Company	Vaccine type	Dosage
AZ	AstraZeneca	Adenovirus vector	2 doses
COM	Pfizer/BioNTech	ARNm	2 doses
JANSS	Janssen	Adenovirus vector	1 dose
MOD	Moderna	ARNm	2 doses

The data from the Ministry of Health of the Government of Spain on the vaccination strategy consist of reports on the evolution of the strategy, i.e. no daily or weekly data on the doses administered are publicly available. Therefore, in this study we use the European COVID-19 vaccination data collected by the European Centre for Disease Prevention and Control. This dataset contains the doses administered per week in each country, grouped by vaccine type and age group. In addition, a distinction is made whether the vaccine corresponds to a first or a second dose. The data source is available in^42^.

**Figure 3:**
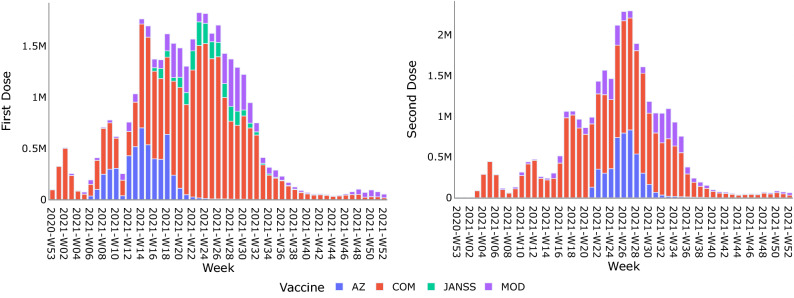
First and second doses of the COVID-19 vaccine given in Spain by week and type of vaccine.

In Fig. 3 we show the weekly evolution of the vaccination strategy considering the type of vaccine, and the first and second doses (without distinguishing by age groups).

**Table 2:** Input for predicting data for days $n+1$ to $n+14$ using ML models.

	Input				
Day predicted	*lag*1	*lag*2	.	*lag*14	Vaccination/mobility/weather data
$n+1$	Cases *n*	Cases $n-1$	.	Cases $n-13$	Data $n-13$
$n+2$	Pred $n+1$	Cases *n*	.	Cases $n-12$	Data $n-12$
$n+3$	Pred $n+2$	Pred $n+1$	.	Cases $n-11$	Data $n-11$
.	.	.	.	.	.
$n+14$	Pred $n+13$	Pred $n+12$	.	Cases *n*	Data *n*

Note the feedback process taking place in the lags column.

**Figure 4:**
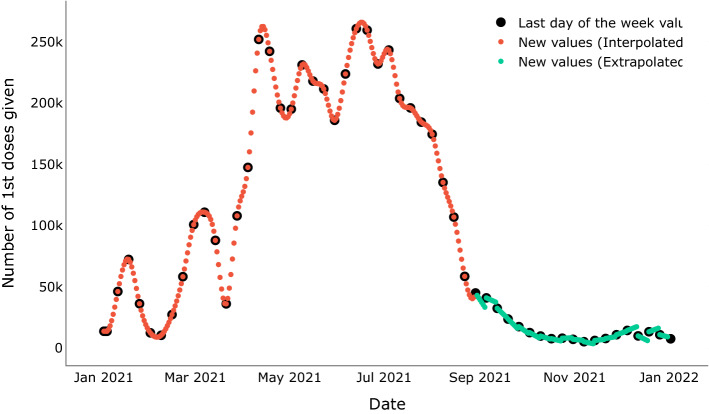
Interpolated and extrapolated values for each day of 2021 for the first dose of the vaccine.

The number of doses administered is given on a weekly basis (i.e. doses administered each week), but we were interested in extrapolating these data to a daily level. As the value of the total weekly doses was not known until the last day of each week, we associated to each Sunday the total value of doses administered that week divided by 7. Then, we had to assign values for the intermediate days. Note that, in order to predict the cases of day *n*, the vaccination, mobility and weather data on day $n-14$ are used (the motivation for this is explained in Subection ML models and in Table 2). Then, in order not to use future data in the test set (we do not know the data from the last available day to *n*), we could not interpolate those values for that part of the data, therefore the implemented process was: we *interpolated* using cubic splines with the known data until August 29th, 2021 (the training set covered up to September 1st, 2021), and from the last known data, we *extrapolated* linearly until the end of that week (when a new observation will be available). That is, if we consider as known days the last day of each week, every time we reach a new known data, we continue the linear extrapolation. The result obtained for the data of the first dose is shown in Fig. 4, where it can be seen which values were known because it was the last day of the week, which were interpolated and which were extrapolated.

Therefore, through a process of interpolation for the train set, and extrapolation for validation and test sets, we associated to each day of 2021 a value for the vaccination data of the first and second doses of COVID-19 vaccine. Figure 4 shows the result corresponding to the first dose, and an analogous process was followed for the second dose.

### Mobility data

In order to assess human mobility we used the data provided by the Spanish National Statistics Institute—in Spanish *Instituto Nacional de Estadística (INE)*—. The data source is available at^43^.

Since 2019 the INE has conducted a human mobility study based on cellphone data. In 2020, during the period corresponding to the state of alarm, and due to the impact of mobility in the COVID-19 pandemic in Spain, this project provided daily information on movements between the 3214 mobility areas that were designed for the original study. For this period, from March 16th to June 20th, the telephone operators provided daily data. Subsequently, due to the continuous waves of the pandemic and the influence of mobility on its evolution, the study continued, but with the publication of weekly data, relative to two specific days of the previous week (Wednesday and Sunday). Information on the study is available at^43^.

Regarding the data collected in this project, we were interested in knowing the flux between different population areas, for which we have areas of residence and areas of destination.

Some important aspects of the data provided by this study are summarized below:

- Cellphones location data were obtained from the three major mobile operators in the country (Orange, Telefónica and Vodafone).
- The area of residence of each cellphone is considered to be the area where it was located for the longest time between 22:00 hours of the previous day and 06:00 hours of the observed day.
- In order to determine the area of destination, all areas (including the residence one) in which the terminal was located during the hours of 10:00 to 16:00 of the observed day were taken. If there were more than one area, the one where the terminal was located the longest time, other than the area of residence, was taken.
- In order to preserve user privacy, whenever the number of observations was less than 15 in an area for a given operator, the result was censored at source. Origin-destination mobility data was then only provided for the areas in which at least one of the three operators pass this threshold.
- As in most of the original data there were available two days for each week, a forward fill was performed when data was not available (i.e. propagating the known values as explained hereinafter).

**Figure 5:**
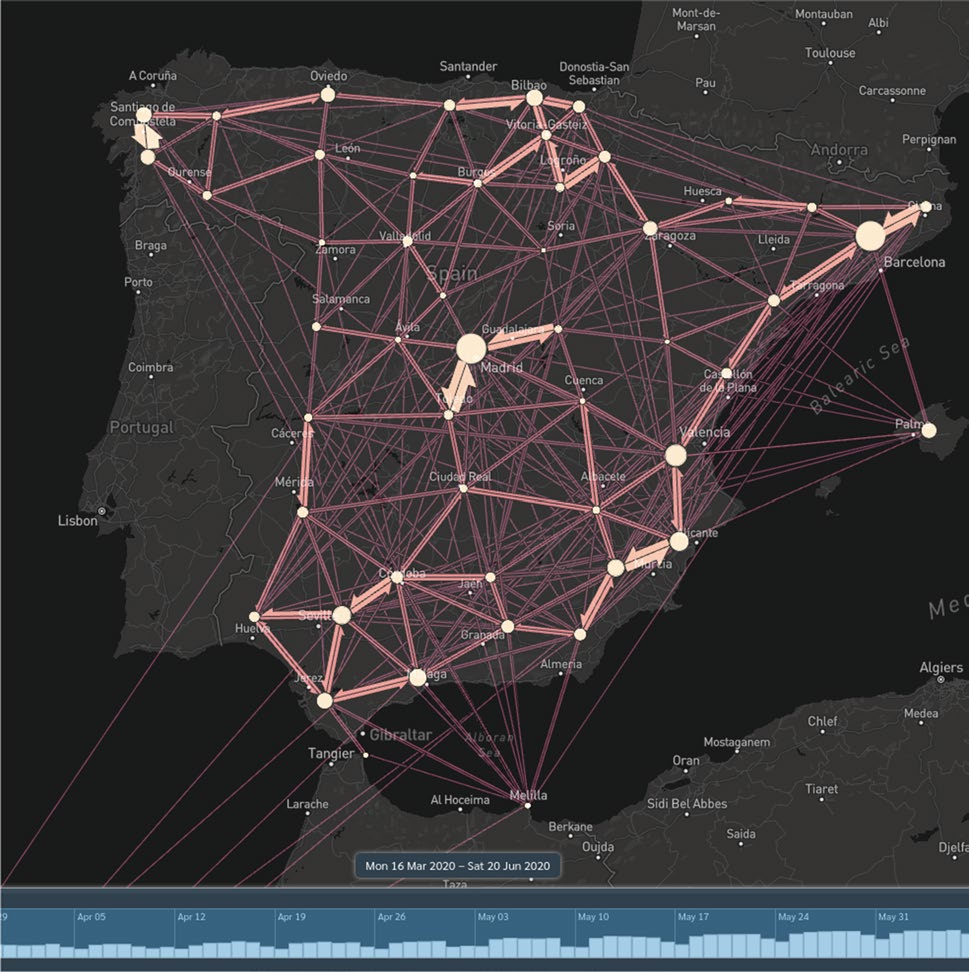
Mobility fluxes in Spain. Arrow size shows inter-province fluxes and dot size shows intra-province fluxes. Visualization has been created with FlowmapBlue (https://flowmap.blue/).

Figure 5 shows a visual representation of the origin-destination fluxes provided by the INE.

Finally, in order to assign a daily mobility value to each autonomous community we implemented the following process. Be $X_i$ each of the *N* autonomous communities considered in the study, $i \in \{1,...,N\}$. The mobility flux assigned to an autonomous community $X_{i}$ on a given day *t* ($F_{X_{i}}^{t}$) is the sum of all the incoming fluxes from the remaining $N-1$ Communities (inter-mobility), that is $f_{X_{j} \rightarrow X_{i}}^{t}$ $\forall j \in \{1,...,N\}$, $j \ne i$, together with the internal flux $f_{X_{i} \rightarrow X_{i}}^{t}$ inside that Community (intra-mobility):

1 $$\begin{aligned} F_{X_{i}}^{t} = \sum _{j=1}^{N} f_{X_{j} \rightarrow X_{i}}^{t} \end{aligned}$$

**Figure 6:**
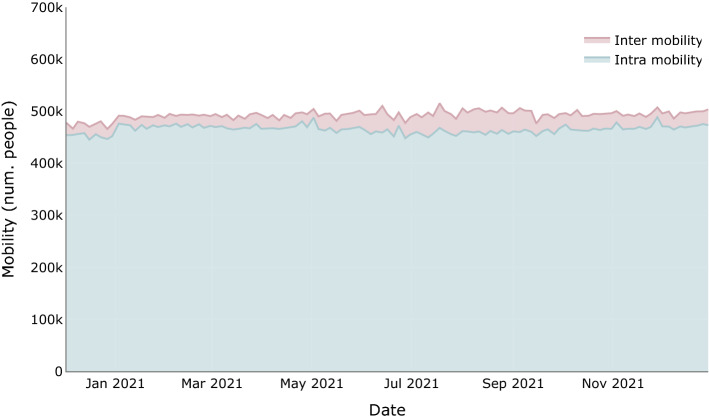
Mobility fluxes in Cantabria, separating the contributions of the two components: intra-mobility (people that move inside Cantabria) and inter-mobility (people that arrive to Cantabria).

When studying the whole country, Spain, the mobility was the sum of the fluxes of all the autonomous communities. Figure 6 shows the temporal evolution of mobility for Cantabria, separating the intra-mobility and inter-mobility components.

**Figure 7:**
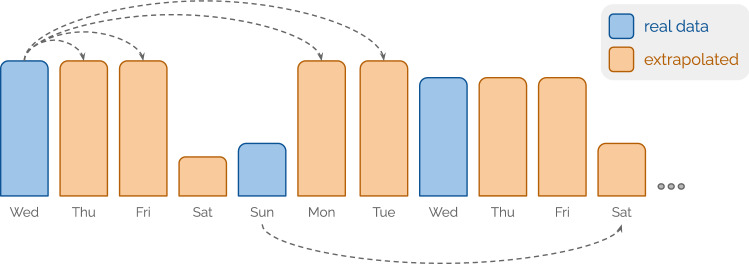
Mobility data processing.

As real mobility data were only published for Wednesdays and Sundays, we implemented the following approach to assign daily mobility values to the remaining days. For each week, we assigned Monday/Tuesday the values of previous Wednesday, Thursday/Friday the values of current Wednesday, and Saturday the value of previous Sunday. The process is shown in Fig. 7.

This approach is based in two key observations: (1) mobility has a strong weekly pattern (higher on weekdays, lower on weekends); (2) We could not directly assign the Wednesday value for all weekdays in the week because that would create an information leak (i.e. on Monday one cannot already know Wednesday mobility); same argument applies also for weekends. Avoiding this information leak is especially important in the test dataset, hence this approach.

### Weather conditions data

As already stated in the Introduction, there is evidence suggesting that temperature and humidity data could be linked to the infection rate of COVID-19. Daily weather data records for Spain, since 2013, are publicly available^44^. However, these data do not include humidity records, therefore we have used precipitation instead. In order to assign a daily temperature and precipitation values to each autonomous community we simply average the mean daily values of all stations located in that autonomous community. In the case of Spain, we take the average of all stations.

As we are mainly interested in seeing if large scale weather trends (mainly seasonal) have and influence of spreading, we have performed a 7-day rolling average of these values (both temperature and precipitations). This also helps reducing the noise in the input data for the models.

### Data limitations

Most of the data limitations that we have faced are of course not exclusive to this paper. But we wanted nonetheless gather them all together so the reader can have a clearer picture of the confidence level on the results here found. Here are some of the limitations we faced while developing this work:

- Incidence data is not always a good proxy for infected people because it relies on the number of diagnostic tests performed. This led to an underestimation of infected people especially at the beginning of the pandemic because the tests were not widely available. Not performing tests on the whole population, just on symptomatic people, also leads to an underestimation of infected people. Holidays may also modify testing patterns.
- Incidence prediction can be reliable usually up to two weeks, but further predictions will be influenced by future data not yet available when making the predictions. These data includes future control measures, future vaccination trends, future weather, etc. Therefore measuring the accuracy of the model for time ranges beyond that limit is not a good assessment of its quality, that is why all results in this work are limited to 14-day forecasts.
- Vaccination data are only available on a weekly basis provided at country level, so fine-grained differences in vaccination progress between regions are lost.
- Spain is a regional state, and each autonomous community is the ultimate responsible for public health decisions, resulting in methodological disparities between administrations when reporting cases.
- Infection data did not report the COVID-19 variants. Therefore models have a limited time-range applicability. Models trained at the beginning of the pandemic will hardly be able to predict the high-rate spreading of the Omicron variant^45^, as it is shown in the “Results” section.
- Mobility data can be misleading, as they do not always equate to risk of infection, because certain activities may suppose more risk of infection than others, regardless of the level of mobility required for each of them. For example, in^46^ it is mentioned that markets and other shopping malls with frequent visitors were areas with high risk of infection (in the case of Wuhan, China), so, in general, mobility to these types of places may suppose a higher exposure to the disease. In addition, we only had the actual data on Wednesdays and Sundays, from which we had to infer the values for the rest of the days.
- The weather value of a region has been taken as the average of all weather stations located inside that region. Despite being a good first approximation, this was obviously not optimal. Stations located near densely populated areas should had greater weight than those located near sparsely populated areas.

## Methods

In this work we have designed an ensemble of models to predict the evolution of the epidemic spread in Spain, specifically ML and population models.

We purposely decided to use population models instead of the classical SEIR models (which are designed to model pandemics) because Spain no longer publishes the data of recovered patients. These daily recoveries (or the daily number of active cases) is crucial in order to estimate the recovery rate, and thus the SEIR basics compartments (**S**usceptible, **E**xposed, **I**nfected, **R**ecovered). As it can be seen in the following equation, the missing data cannot be inferred from available data, so the data on the daily recovered were not available:

$$\begin{aligned} {Confirmed} = {Active} + {Recovered} + {Deceased} \end{aligned}$$

In this study we used a training set to train the ML models and fit the parameters of the population models. In order to make the ensemble, the predictions of each model for the test set are weighted according to the root-mean-square error (RMSE) in the validation set.

### Computing environment

The computations were performed using the DEEP training platform^47^. Also, this work was implemented using the Python 3 programming language^48^. In particular, the following additional libraries and versions were used: scikit-learn^49^ version 0.24.2, scipy^50^ version 1.7.1, pandas^51^ version 1.3.3, numpy^52^ version 1.21.2, and plotly^53^ version 5.3.1. Additionally flowmap.blue^54^ was used to visualize flow maps.

### Models definition

#### Population models

Population models are mathematical models applied to the study of population dynamics. The classic application of this kind of models is to analyze and predict the growth of a population^55^. However, there are numerous applications in other fields, from animal growth^56^, tumor growth^57^, evolution of plant diseases^58^, etc. In addition, several works use this type of model to try to predict the future trend of COVID-19 cases, as exposed in section “Related work”.

Specifically in this study, we used the following four models.

- **Gompertz model** is a type of mathematical model that is described by a sigmoid function, so that growth is slower at the beginning and at the end of the time period studied. It is used in numerous fields of biology, from modeling the growth of animals and plants to the growth of cancer cells^59^. Be *p*(*t*) the population at time t, then, the ordinary differential equation (ODE) which defines the model is given by:
  2 $$\begin{aligned} \frac{\partial p}{\partial t} = ap(t) -bp(t)log(p(t)) \end{aligned}$$
  And its explicit solution:
  $$\begin{aligned} {p(t) = e^{\frac{a}{b}+c e^{-bt}}} \end{aligned}$$
- **Optimized parameters**: once we have the explicit solution for the ODE of the model, we need to estimate the three parameters involved: *a*, *b* and *c*. To do so, we follow the process described in the last section of the Supplementary Materials (Explicit solution of the ODE of the Gompertz model and estimation of the initial parameters). When we get an initial estimation for *a*, *b* and *c*, these parameters are optimized using the explicit solution of the ODE and the known training data. Specifically in our study we have used the sum of squares of the error for this purpose.
- **Implementation**: for the optimization of parameters from the initial estimation, fmin function from the optimize package of scipy library^50^ was used.
- **Logistic model** was introduced by Verhulst in 1838^60^, and establishes that the rate of population change is proportional to the current population *p* and $K-p$, being *K* the carrying capacity of the population. Thus, be *a* the constant of proportionality, and $b =\frac{a}{K}$, the ODE that defines the model it is given by:
  3 $$\begin{aligned} \frac{\partial p}{\partial t} = ap(t)-bp^{2}(t) \end{aligned}$$
  And the explicit solution:
  $$\begin{aligned} {p(t) = \frac{1}{c e^{-at}+\frac{b}{a}}} \end{aligned}$$
  Again it is necessary to calculate some initial parameters, which are optimized as in the case of the Gompertz model) *a*, *b* and *c*.
- **Optimized parameters**: *a*, *b* and *c*, first estimated following an analogous process to that of the Gompertz model.
- **Implementation**: for the optimization of the initial parameters fmin function from the optimize package of scipy library^50^ has been used.
- **Richards model** is a generalization of the logistic model or curve^61^, introducing a new parameter *s*, which allows greater flexibility in the modeling of the curve. It is defined by the following ODE:
  4 $$\begin{aligned} \frac{\partial p}{\partial t} = \frac{a}{s}p(t)\left( 1-\left( \frac{p(t)}{p_{\infty }}\right) ^{s}\right) \end{aligned}$$
  And the explicit solution:
  $$\begin{aligned} {p(t) = \frac{1}{\left( c e^{-at}+\frac{1}{(p_{\infty })^{s}}\right) ^{\frac{1}{s}}}} \end{aligned}$$
  Note that if $s = 1$ we are considering the logistic model:
  $$\begin{aligned}&\underbrace{\frac{\partial p}{\partial t} = a p(t)\left( 1-\frac{p(t)}{p_{\infty }} \right) }_{\text {ODE Richards Model (s=1)}} = a p(t) - \frac{a}{p_{\infty }} p^{2}(t) \overset{p_{\infty } = \frac{a}{b}}{\Longrightarrow } \\&\overset{p_{\infty } = \frac{a}{b}}{\Longrightarrow } \underbrace{\frac{\partial p}{\partial t} = ap(t)-bp^{2}(t)}_{\text {ODE Logistic Model}} \end{aligned}$$
- **Optimized parameters**: in view of the above, we considered as the initial values for *a*, *b* and *c* those optimized parameters after training the logistic model and $s=1$.
- **Implementation**: for the optimization of the initial parameters fmin function from the optimize package of scipy library^50^ was used.
- **Bertalanffy model** or the Von Bertalanffy growth function (VBGF) was first introduced and developed for fish growth modeling since it uses some physiological assumptions^62,63^. However, some studies show its possible applications to other types of scenarios, adapting its parameters to be used as a model for population modeling^64^. It is therefore reasonable to study the applicability of this model to the evolution of COVID-19 positive cases, as is done in^65^. The general formulation of the function is given by the following ODE^66^:
  5 $$\begin{aligned} \frac{\partial p}{\partial t} = a p^{m}(t) + b p^{n}(t) \end{aligned}$$
  Although numerous studies focus only on an appropriate choice of *n* and *m* values^67^, as we seek to test the fit of this model, we take two standard parameters $n=1$ (which is widely assumed^68^) and $m=3/4$ as proposed in^69^. Thus, the explicit solution of the ODE is:
  $$\begin{aligned} {p(t) = \left( \frac{a}{b}+ce^{\frac{-bt}{4}}\right) ^{4}} \end{aligned}$$
- **Optimized parameters**: *a*, *b* and *c* first estimated following a process analogous to that of the Gompertz model.
- **Implementation**: for the optimization of the initial parameters fmin function from the optimize package of scipy library^50^ has been used.

**Figure 8:**
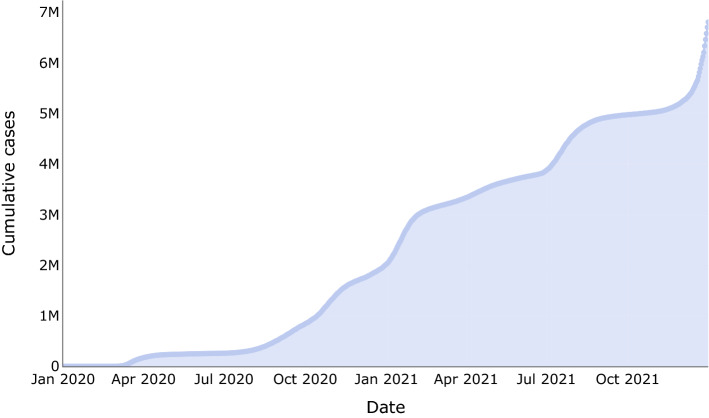
Cumulative COVID-19 confirmed cases in Spain since the start of the pandemic.

The main motivation to use this type of models was the shape of the curve of the cumulative COVID-19 cases. Figure 8 shows the cumulative cases in Spain. It can be seen that many sections of the curve follow a sigmoid shape, which can be modeled, as we have shown, with the previously presented models. Thus, we can take a relatively short period of time (e.g. 30 days), prior to the days we want to predict and apply the previous population models optimizing their parameters to adapt to the shape of the curve and make new predictions.

#### Machine learning models

After training several ML models and testing their predictions on a validation set and a test set, we reduced the set of models to the following four: Random Forest, k-Nearest Neighbours (kNN), Kernel Ridge Regression (KRR) and Gradient Boosting Regressor. All the models under study minimize the squared error of the prediction (or similar metrics).

The parameters of each model were optimized using stratified 5-folds cross-validated grid-search, implemented with GridSearchCV from sklearn^49^.

- **Random Forest** is an ensemble of individual decision trees, each trained with a different sample (bootstrap aggregation)^70^. This type of model is a bagging technique, and the different individual classifiers that it uses (decision trees) are trained without interaction between them, in parallel.
- **Optimized parameters**: the maximum depth of the individual trees, and the number of estimators, i.e. individual trees in the forest.
- **Implementation**: RandomForestRegressor class from sklearn^49^.
- **k-Nearest Neighbours (kNN)** is a supervised learning algorithm, and is an example of instance-based learning. The basic idea of this model is very simple: given a distance (e.g. Euclidean, Manhattan or Hamming distance), the *k* points of the train set that are closest to the test input *x* with respect to that distance are searched, to infer what value is assigned to that input^71^.
- **Optimized parameters**: number of neighbors (*k*)
- **Implementation**: KNeighborsRegressor class from sklearn^49^.
- **Kernel Ridge Regression (KRR)** is a simplified version of Support Vector Regression (SVR). In short, this technique combines Ridge regression (LS and normalization with $l_{2}$ norm), and the kernel trick. For details on this technique, see e.g.^72^.
- **Optimized parameters**: $\alpha$ and $\gamma$ (see^73^).
- **Implementation**: KernelRidge class from sklearn^49^ (with an rbf kernel).
- **Gradient Boosting Regressor** is a boosting-type (combines weak learners into a strong learner) algorithm for regression^74^. In particular, it is an ensemble of individual decision trees trained sequentially.
- **Optimized parameters**: learning rate and the number of estimators (i.e. the number of individual trees considered).
- **Implementation**: XGBRegressor class from the XGBoost optimized distributed gradient boosting library^75^.

### Model inputs and outputs

In the following sections the technicalities of what inputs are needed and how outputs are generated for each kind of model family are discussed. In particular, in this work we generated 14-day forecasts with both population and ML models.

#### Population models

Population models are trained with the daily accumulated cases of the 30 days prior to the start date of the prediction. Once fitted with these data, the model returns the subsequent days prediction (14 days in this case).

As already stated, population models use the accumulated cases (instead of raw cases) because it intermittently follows a sigmoid curve (cf. Figure 8) that these models are especially designed to fit. It should additionally be stressed that population models do not use the rest of the variables (such as mobility, vaccination, etc) that are included in ML models.

#### Machine learning models

The process of generating time series predictions with ML models is recurrent. One generates the prediction for the first day ($n+1$), then one feeds back that prediction back to the model to generate $n+2$, and so on until reaching $n+14$. In order to generate a prediction of the cases at $n+1$ the models use the cases of the last 14 days (lag_1-14_) as well as the data at $n-14$ for the other variables (mobility, vaccination, temperature, precipitation). We only use $n-14$ and not more recent data (*n*, ..., $n-13$) because these variables have delayed effects on the pandemics evolution.

In the case of vaccination data, the main motivation to include this lag is that the COVID-19 vaccines manufactured by Pfizer, Moderna and AstraZeneca are considered to protect against the disease two weeks after the second dose. With the Janssen vaccine, this value rises to four weeks after the administration of one dose. However, in order to unify criteria, since in this study the data are not distinguished by type of vaccine administered, a two-week delay was considered (see^76^).

In the case of mobility data, in^77^ it is mentioned that scenarios with a lag of two and three weeks of mobility data and COVID-19 infections are considered for the statistical models. Additionally^78^ found that decreases in mobility were said to be associated with substantial reductions in case growth two to four weeks later.

Finally, with respect to the weather data, in^79^ the authors conclude that the best correlation between weather data and the epidemic situation happens when a 14 days lag is considered. It should be noted that we have taken a 7-day rolling average to reduce the noise and capture the trend in temperature and precipitation (for further details on the weather data pre-processing see section “Weather conditions data”).

The input selection for the recurrent prediction process is illustrated in Table 2. Note that the data were standardized (by removing the mean and scaling to unit variance) using StandandarScaler from the preprocessing package of the sklearn Python library^49^.

Regarding the input variables of the ML models, we tested different configurations depending on the input data included. Figure 2 of Supplementary Materials shows the results obtained with different input configurations. After performing different tests, we decided to analyze the four scenarios exposed in Table 3.

**Table 3:** Input data for ML models.

Input	Scenarios			
	1	2	3	4
Cases	$\checkmark$	$\checkmark$	$\checkmark$	$\checkmark$
Vaccination		$\checkmark$	$\checkmark$	$\checkmark$
Mobility			$\checkmark$	$\checkmark$
Weather				$\checkmark$

### Metrics and model ensemble

We used the mean absolute percentage error (MAPE) and the root mean squared error (RMSE) to evaluate the quality of the predictions. The error assigned to a single 14-day forecast is the mean of the errors for each of the 14 time steps.

When aggregating predictions of both types of models, we considered the models equally, independently of the type (ML or population) they belong to. Nevertheless, we provide disaggregated results for each type to highlight the qualitative differences in their predictions.

We followed several possible strategies to create the ensemble of the models:

- **Mean** prediction of all the models.
- **Median** value of the prediction of all models.
- **Weighted average (WAVG)** prediction, where the weight given to each model is the inverse of the RMSE of that particular model on the validation set (cf. section “Data” for the date ranges of the different splits). That is, the better the performance of a model, the higher the weight assigned to the model.

## Results and discussion

### Results

In this section, we focus on the results and analysis of the models trained on Spain as a whole. We, nevertheless, provide in the Supplementary Materials (Analysis by autonomous community) a similar analysis for the 17 Spanish autonomous communities.

Tables 4 and 5 show the MAPE and RMSE performance for the test set. Columns encode inputs provided to the ML models (cf. Table 3) while rows show the different aggregation methods (cf. section “Metrics and model ensemble”) applied to different subsets of models (ML, Pop, All). Additional plots with model-wise errors are provided in the Supplementary Materials (Fig. 5).

**Table 4:** MAPE obtained in each scenario according to each form of aggregation, for the Spain case in the test split.

Aggregation		Scenario			
		1	2	3	4
Mean	ML	0.5993	0.5571	0.5166	**0.5052**
	Pop	0.5210	–	–	–
	All	**0.3424**	0.3501	0.3442	0.3470
Median	ML	0.6224	0.5060	**0.4610**	0.4688
	Pop	0.5007	–	–	–
	All	**0.3120**	0.3352	0.3515	0.3932
WAVG	ML	0.5831	0.5088	0.4427	**0.4219**
	Pop	0.4954	–	–	–
	All	**0.3090**	0.3375	0.3363	0.3411

Lowest error values are in [bold].

**Table 5:** RMSE obtained in each scenario according to each form of aggregation, for the Spain case in the test split.

Aggregation		Scenario			
		1	2	3	4
Mean	ML	**9510.0**	10121.3	10015.4	10032.7
	Pop	10006.2	–	–	–
	All	**9314.0**	9718.4	9700.4	9728.0
Median	ML	**9508.8**	10069.2	9935.3	9921.0
	Pop	9537.2	—	–	–
	All	**9235.9**	9693.7	9694.1	9783.1
WAVG	ML	**9506.9**	9857.5	9602.6	9624.1
	Pop	9713.0	–	–	–
	All	**9201.0**	9481.7	9427.9	9471.4

Lowest error values are in [bold].

**Figure 9:**
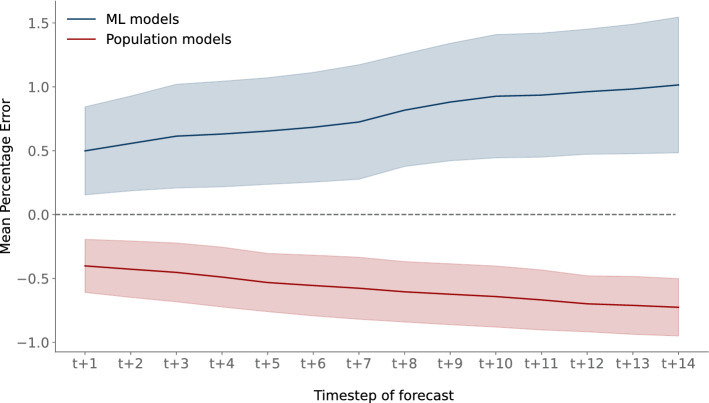
MPE for each time step of the forecast, grouped by model family, for the Spain case in the validation split. Shades show the standard deviation between models of the same family. ML models are trained in Scenario 4.

Focusing on the MAPE (Table 4), one can notice (comparing column-wise) that the WAVG performs better than median aggregation which in turn performs better than mean aggregation. When comparing (row-wise) different ML models (ML rows) we see that adding more variables generally leads to a better performance. Nevertheless, when we average these ML models with population models (All rows), adding more variables seems to be detrimental. The answer to this apparent contradiction comes from looking at the relative error for each model family. For this, in Fig. 9, we plot the Mean Percentage Error (MPE) (i.e. same as MAPE but without taking the absolute value) obtained for each of the 14 time steps in the validation set. We clearly see that ML models tend to overestimate, while population models tend to underestimate. This means that when we combine both model families the positive and negative errors cancel out, leading to a better overall prediction. However, this entails that if we improve ML models alone (by adding more variables in this case), when we combine them with population models the errors end up not cancelling as before. This explains the apparent contradiction that better ML models do not necessarily lead to better overall ensembles. It is worth noting than in Fig. 9, both model family errors increase as the forecast time step does. But this increase is not evenly distributed, as ML models degrade faster than population models, while their performance is on par at shorter time steps.

**Figure 10:**
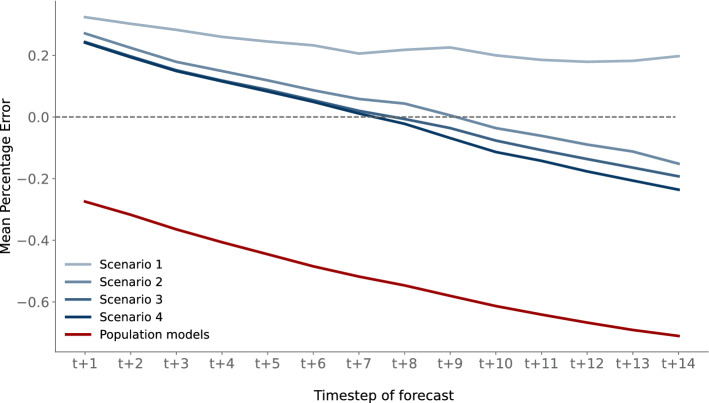
MPE for each time step of the forecast, grouped by model family, for the Spain case in the test split. ML models are shown for the 4 different scenarios.

The previous analysis on the validation set corresponds to a stable phase in COVID spreading, enabling us to clearly identify the over/underestimate behaviour and the performance degradation in both families. The test set however is dominated by an exponential increase in cases due to the sudden appearance of the Omicron variant around mid-November (cf. Fig. 1). The patterns detected in the validation set still hold, but they are not as straightforward to see. In Fig. 10 we show the MPE error in the test set, both for population models and ML models trained on several scenarios.

Now, due to the sudden increase in cases, ML models start overestimating, but as the time step increases they end up underestimating. This explains why Scenario 3 has sometimes lower MAPE (cf. median aggregation and ML row in Table 4) than Scenario 4, which has more input variables. While it should have worse error, the fact that ML models end up underestimating means that Scenario 3 underestimates less than Scenario 4, giving sometimes (depending on the aggregation method) a better overall prediction.

Regarding population models, they still underestimate but much more severely than ML models, as expected from the previous analysis on the validation set. Paired with the progressive underestimation of ML models, this means the ensemble tends to be worse when more input variables are added (because ML models with less input variables underestimate less), as seen in the All rows in Table 4.

Finally, we provide in Fig. 4 of Supplementary Materials a similar plot but subdividing the test set into a stable (no-omicron) and an exponentially increasing (omicron) phase, where we make the same analysis performed with the validation set.

For RMSE (Table 5), comparing column-wise, one still sees that each aggregation method improves on the previous one. But surprisingly, comparing row-wise on ML rows, we notice that the results go inversely than MAPE results. That is, adding more variables to the ML models leads to worse performance.

Again, this can be explained if we take a closer look at the propagation dynamics during the test split. Note that, as observed in Fig. 1, since mid-November we observe an exponential increase of cases which corresponds to the spread of the Omicron variant.

In Fig. 3 of Supplementary Materials, we subdivide the test results into 2 splits (no-omicron, omicron). We see that inside each split, RMSE and MAPE follow the same trend and the contradiction disappears. For the no-omicron phase, the best ML scenario is always the one with all the inputs. For the omicron phase, both MAPE and RMSE suggest that the best ML scenario is the one just using cases as input variable. This may be due to the importance of the first lags in capturing the significant growth of daily cases. In the full test split, the contradiction appeared because RMSE gives more weight to dates with higher errors (i.e. the omicron phase), while MAPE weights are evenly distributed.

This analysis suggests that the model is not robust to changes of COVID variant. When it predicts the same variant that it was trained on, the model knows how to make good use of all inputs. But when a new variant appears, the spreading dynamics changes, and therefore additional inputs just confuse the model, which prefers to rely solely on the cases. Changes in dynamics include facts like Omicron being more contagious (that is, same mobility leads to more cases than with the original variant) and being more resistant to vaccines (that is, same vaccination levels leads to more cases than with the original variant)^80^.

**Figure 11:**
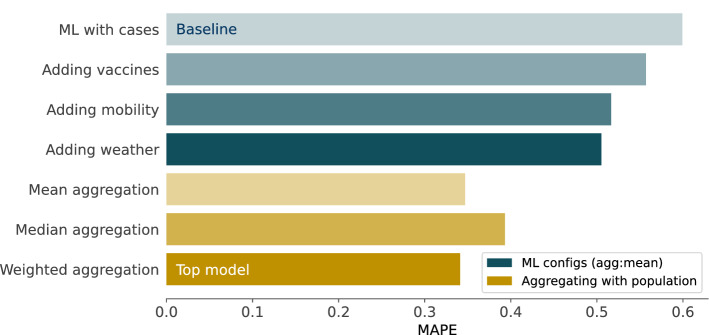
Cumulative improvements for the Spain case in the test split. We color separately (1) improvements made on ML models by adding more inputs (aggregating always with mean), (2) improvements made when aggregating the ML models (with full inputs) with population models with different aggregation methods.

Finally, as a visual summary of Table 4 results, we show in Fig. 11 how starting with the most basic ensemble (only ML models trained with cases), one can progressively add improvements (more input variables, better aggregation methods), until achieving the best performing ensemble (ML models trained with all variables and aggregated with population models). The degraded performance with the median aggregation is due to the fact, as discussed earlier, that while ML models improved, the total aggregation with population models happened to be worse.

### Interpretability of ML models

The interpretability of ML models is key in many fields, being the most obvious example the medical or health care field^81^. Understanding the reasons why a model based on artificial intelligence techniques makes a prediction helps us to understand its behavior and reduce its black box character^82^. For this purpose, in this work we have used the SHapley Additive exPlanation (SHAP) values^83^.

SHAP values are used to estimate the importance of each feature of the input characteristics space in the final prediction. The idea is to study the predictions obtained when a feature is removed or added from the model training. Specifically, the final contribution of input feature *i* is determined as the average of its contributions in all possible permutations of the feature set^82^. Having a positive/negative SHAP value for input feature *i* on a given day *t* means that feature *i* on day *t* contributed to pushing up/down the model prediction on day *t* (with respect to the expected value of the prediction, computed across the whole training set).

**Figure 12:**
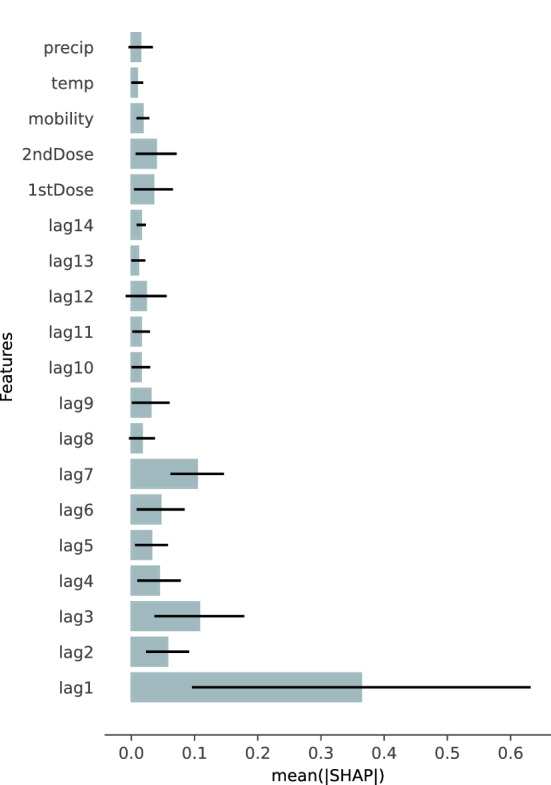
Mean absolute SHAP values (normalized). Error bars show the standard deviation across all the ML models.

In Fig. 12, we plot the importance of the different features: how much the model relies on a given feature when making the prediction. This importance is computed taking the mean value (across the full dataset) of the absolute value (it does not matter whether the prediction is downward or upward) of the SHAP value. This is done feature wise and averaging the 4 ML models studied (cf. section “Interpretability of ML models”): Random Forest, Gradient Boosting, k-Nearest Neighbors and Kernel Ridge Regression.

We see that the features of the lags of the cases, especially the first lags, have the biggest impact on the predictions. As expected, the larger the lag, the lower the importance of that feature (i.e. more recent the data, the more it matters), with some noisiness in the decrease (e.g. $lag_3$, $lag_7$).

At a first glance one might think that non-cases features (vaccination, mobility and weather), do not matter much in comparison to the first lags of the cases. This view is obviously biased. The first lags give a rough estimate of future cases (i.e. future cases are roughly equal to present cases), but the remaining features, while smaller in absolute importance, are crucial to refine the rough estimate upwards or downwards. And this is precisely why we saw that adding more variables always reduced the MAPE of ML models (cf. Table 4).

In Figs. 6 and 7 of the Supplementary Materials we provide a more in depth overview of the contribution of each feature.

For the case lags, we see that the positive slope in the $lags_{1-7}$ shows that higher lag values correlate with higher predicted cases, which is obviously expected. For $lags_{8-13}$, this trend is inverted, meaning that higher lag values correlate with lower predicted cases. This is obviously counter-intuitive and we do not have a clear conclusion about why this might be happening, but it is possibly due to some complex interaction between several features. In $lag_{14}$ the trend goes back to normal again, suggesting that the model is following some weekly pattern in the lags (as $lag_7$ was also abnormally high) which might be reflecting the moderate weekly pattern we saw in Fig. 2.

For non-cases features, we see that:

- Mobility is not strongly correlated with predicted cases. This is possibly due to the fact that mobility is misleading: when cases grow fast, mobility is restricted, but cases keep growing due to inertia.
- Precipitation is not correlated with predicted cases (probably because precipitation is not a good proxy for humidity).
- Higher temperatures are correlated with lower predicted cases as expected (see, for instance,^10^).
- Higher number of first vaccine dose are moderately correlated with lower predicted cases as expected, while second dose does not show mayor correlations. Although unexpected, this lack of negative correlation (more vaccines, lower cases) can be explained by the fact that vaccination efforts tend to increase during peaks in cases, therefore, as with mobility, cases keep growing due to inertia despite vaccination efforts.

### What ended up not working

Every paper that does not contain its *counterpaper* should be considered incomplete^84^. Therefore we dedicate this section to briefly describe some of the aspects that we have considered, but that ended up not being included in the final model. We also hope to provide, when possible, some insights as for why they did not improve accuracy as expected.

#### Input pre-processing

When deciding the mobility/vaccination/weather lags, we tested in each case a number of values based on the lagged-correlation of those features with the number of cases. In the end, the correlation was not a good predictor of the optimal lag, so we decided to go with the community standard values (14 day lags, cf. section “Data”). In addition, we tried to include a weekday variable (either in the [1, 7] range or in binary as weekday/weekend) to give a hint to the model as when to expect a lower weekend forecast. This did not end up working, possibly due to the fact that the weekly patterns in the number of cases are often relatively moderate compared to the large variations in cases throughout the year (cf. Fig. 2).

When we fixed the inputs we were going to use, we tested a number of pre-processing techniques that did not improve the model performance. Among those:

- We performed a 7-day rolling average of the mobility to smooth the weekly mobility patterns.
- We provided accumulated vaccination instead of raw vaccination. Using cumulative vaccines made more sense than using new vaccines, because we would not expect a sudden increase in cases if vaccination was to be stopped for one week, especially if a large portion of the population is already vaccinated.
- In addition to the raw features, we added the velocity and acceleration of each feature (cases/mobility/vaccination), to give a hint to the models about the evolution trend of each feature.

In the end, all these *a priori* sensible pre-processing techniques might not have worked because, as we saw in section “Interpretability of ML models”, the correlations between these variables and the predicted cases was not strong enough and their absolute importance was small compared with cases lags to be distorted by noise.

Finally, regarding the selection of the four scenarios studied, in addition to the configurations discussed above which did not perform successfully, we have tested the seven possible combinations of cases and variables, namely: ***cases + vaccination***, *cases + mobility*, *cases + weather*, ***cases + vaccination + mobility***, *cases + vaccination + weather*, *cases + mobility + weather* and ***cases + vaccination + mobility + weather***. After performing these tests, we decided to analyse the scenarios shown in Table 3 because they were the ones that provided the best results.

In Fig. 2 of Supplementary Materials we provide a scatter plot with the performance of these additional experiments.

#### Output structure

Regarding the generation of the forecasts, we generated a single 14-day forecast but it produced substantially worse results. Generating 1-step forecasts and feeding them back to the model, as we finally did, allowed the model to better focus and remove redundancies in the predicting task.

#### Aggregation methods

As an additional aggregation method we tried *stacking*^85^, where a meta ML model (here, a simple Random Forest) learns the optimal way to aggregate the predictions of the ensemble of models. This meta-model is trained on the validation set (to not favour models that over fit the training set). In order to have a single meta-model to aggregate both population and ML models, we fed the meta-model with just the predictions of each model for a single time step of the forecast. In other settings, meta-models use both inputs and predictions, but this was not feasible in our case where inputs varied for population and ML models, and across ML scenarios.

In the end, stacking did not improve results, in most cases performing even worse than the simple mean aggregation. This is possibly due to the small size of the validation set, which makes it difficult to learn a meaningful meta-model. Variations of this setup included (1) training a different meta-model for each forecast time step (same performance as single meta-model setup); (2) feeding the meta-model all 14 time steps (worse performance due to noise added by redundant information).

We also tried to a variation of the weighted average in which we weighted models based on their performance on the validation set, but weighting *each time step separately*. In principle, this should work better than the standard weighting as it learns to give progressively less weight to models whose forecast degrades more rapidly (that is ML models, cf. Fig. 9). In practice it did not show an unequivocal superior performance over the standard weighting, performing in some cases better, in others worse. This is possibly due to the fact that in both setups, weights are computed based on the performance on the validation set, which is relatively small. Therefore one expects that, with more validation data available, the noise cancels out. For the time being, given that the two methods showed similar performance, we decided to favour the simpler approach.

## Conclusions

In this work we have evaluated the performance of four ML models (Random Forest, Gradient Boosting, k-Nearest Neighbors and Kernel Ridge Regression), and four population models (Gompertz, Logistic, Richards and Bertalanffy) in order to estimate the near future evolution of the COVID-19 pandemic, using daily cases data, together with vaccination, mobility and weather data. Specifically, our proposal is to use the two families of models to obtain a more robust and accurate prediction.

With regard to the population models, it should be noted that we have used them as an alternative to the compartmental ones because all the data necessary to construct a SEIR-type model were not available for the case of Spain. Despite their simplicity, we have successfully made an ensemble together with ML models, improving the predictions of any individual model. We are currently not aware of any work including an ensemble of both ML and population models for epidemiological predictions.

In addition, we found that, when more input features were progressively added, the MAPE error of the aggregation of ML models decreased in most cases. We also saw that this improvement did not necessarily reflected on a better performance when we combined them with population models, due to the fact that ML models tended to overestimate while population models tended to underestimate. Therefore, improving ML models alone can unbalance the ensemble, leading to worse overall predictions. Following this analysis, we found that ML models performance degraded when new COVID variants appeared. This, in turn, explains why the RMSE error seemed to deteriorate when adding more input features, seemingly contradicting the MAPE error. When accounting for the change in COVID variant, the metrics agreed again.

Finally, we computed the SHAP values obtained for each of the 4 ML models to assess the importance of each feature in the final prediction. As expected, this highlighted the importance of recent cases when predicting future cases. Among non-cases features, vaccination and mobility data proved to have significant absolute importance, while lower temperatures showed to be correlated with lower predicted cases. All in all, despite relatively minor absolute importance, non-case features (vaccination, mobility and weather) have proven to be crucial in refining the predictions of ML models.

The conclusion of this work is that an ensemble of ML models and population models can be a promising alternative to SEIR-like compartmental models, especially given that the former do not need data from recovered patients, which is hard to collect and generally unavailable.

## Challenges and future directions

We foresee several lines to build upon this work. Firstly, adding more and better variables as inputs to the ML models; for example, introducing data on social restrictions (use of masks, gauging restrictions, etc), on population density, mobility data (type of activity, region’s connectivity, etc), or more weather data such as humidity. Second, regarding the types of models, we will explore deep learning models, such as Recurrent Neural Networks (to exploit the time-dependent nature of the problem), Transformers (to be able to focus more closely on particular features), Graph Neural Networks (to leverage the network-like spreading dynamics of a pandemic) or Bayesian Neural Networks (to quantify uncertainty in the model’s prediction). All this future work will improve the robustness and explainability of the model ensemble when predicting daily cases (and potentially other variables like Intensive Care Units), both at national and regional levels.

## Supplementary Information

Supplementary Information.

## Data Availability

The datasets generated and/or analyzed during the current study are available as follows: data on daily cases confirmed by COVID-19 are available from the Carlos III Health Institute—in Spanish *Instituto de Salud Carlos III (ISCIII)*— at https://cnecovid.isciii.es/covid19^40^. Vaccination data ire avalable from the Ministry of Health of the Government of Spain at https://www.ecdc.europa.eu/en/publications-data/data-covid-19-vaccination-eu-eea^42^. Human mobility data are available from Spanish National Statistics Institute —in Spanish *Instituto Nacional de Estadística (INE)*— at https://www.ine.es/covid/covid_movilidad.htm^43^. Daily weather data records for Spain, since 2013, are publicly available at https://datosclima.es/index.htm^44^.
