# Supplementary material for: Forecasting COVID-19 spreading through an ensemble of classical and machine learning models: Spain’s case study
Source: Sci Rep. 2023 Apr 25;13:6750. doi: 10.1038/s41598-023-33795-8 (PMC10127188; doi:10.1038/s41598-023-33795-8)
Supplement: Supplementary file 1 — Supplementary Information. [file 41598_2023_33795_MOESM1_ESM.pdf]

Supplementary materials: Forecasting COVID-19 spreading through an ensemble of classical and machine learning models: Spain's case study

Ignacio Heredia Cacha[1], Judith Sáinz-Pardo Díaz[1], María Castrillo Melguizo[1], and Álvaro López García[1*]

[1]Instituto de Física de Cantabria (IFCA), CSIC-UC , Avda. los Castros s/n. 39005 - Santander (Spain)
[*]aloga@ifca.unican.es

ABSTRACT

This document contains the supplementary materials for the manuscript "Forecasting COVID-19 spreading through an ensemble of classical and machine learning models: Spain's case study".

Analysis by Autonomous Community

In the following figure the MAPE obtained considering scenario 4 (the most complete) for the machine learning models, and the 3 forms of aggregation: mean, median and weighted mean, is shown for the 17 Spanish autonomous communities.

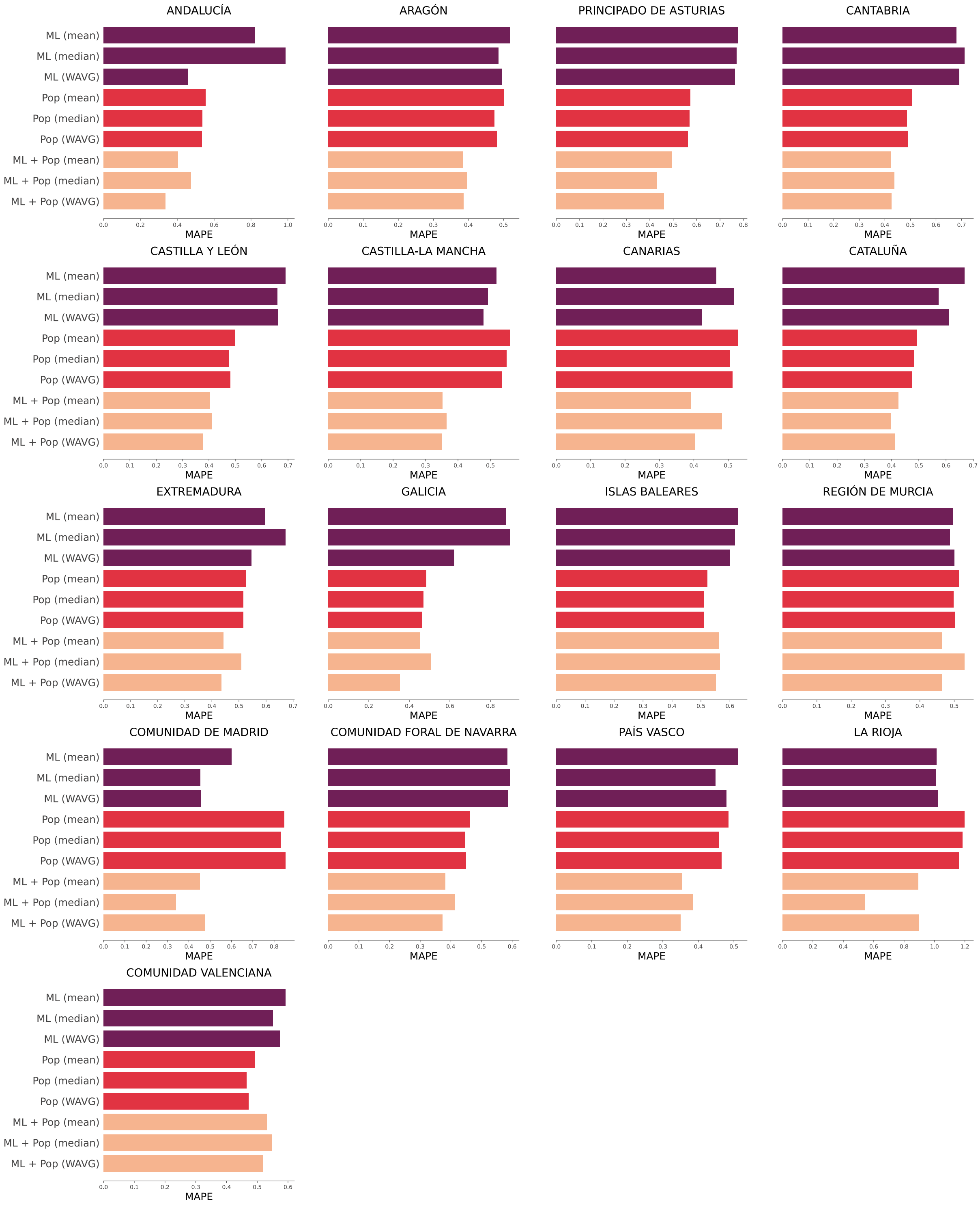

Supplementary figure 1. Summary of the MAPE obtained for the cases of the 17 Spanish autonomous communities with the different models. ML models are trained in Scenario 4.

The main aspect to highlight in the previous figure is that in most cases it is still true that the ensemble of models (with any of the three forms of aggregation), manages to improve the MAPE results of the two families of models individually. This particularly clear in Autonomous Communities like Aragón, Cantabria, Castilla y León, Castilla La Mancha, Cataluña or País Vasco.

Regarding whether Machine Learning or population models obtain better results, it is observed that this changes depending on the autonomous community. In some of them, ML models always obtain better MAPE than population ones (e.g. Madrid, Castilla La Mancha, and La Rioja), while in others cases population models obtain better results than ML ones (e.g. Cantabria, Castilla y León and Cataluña).

Supplementary materials

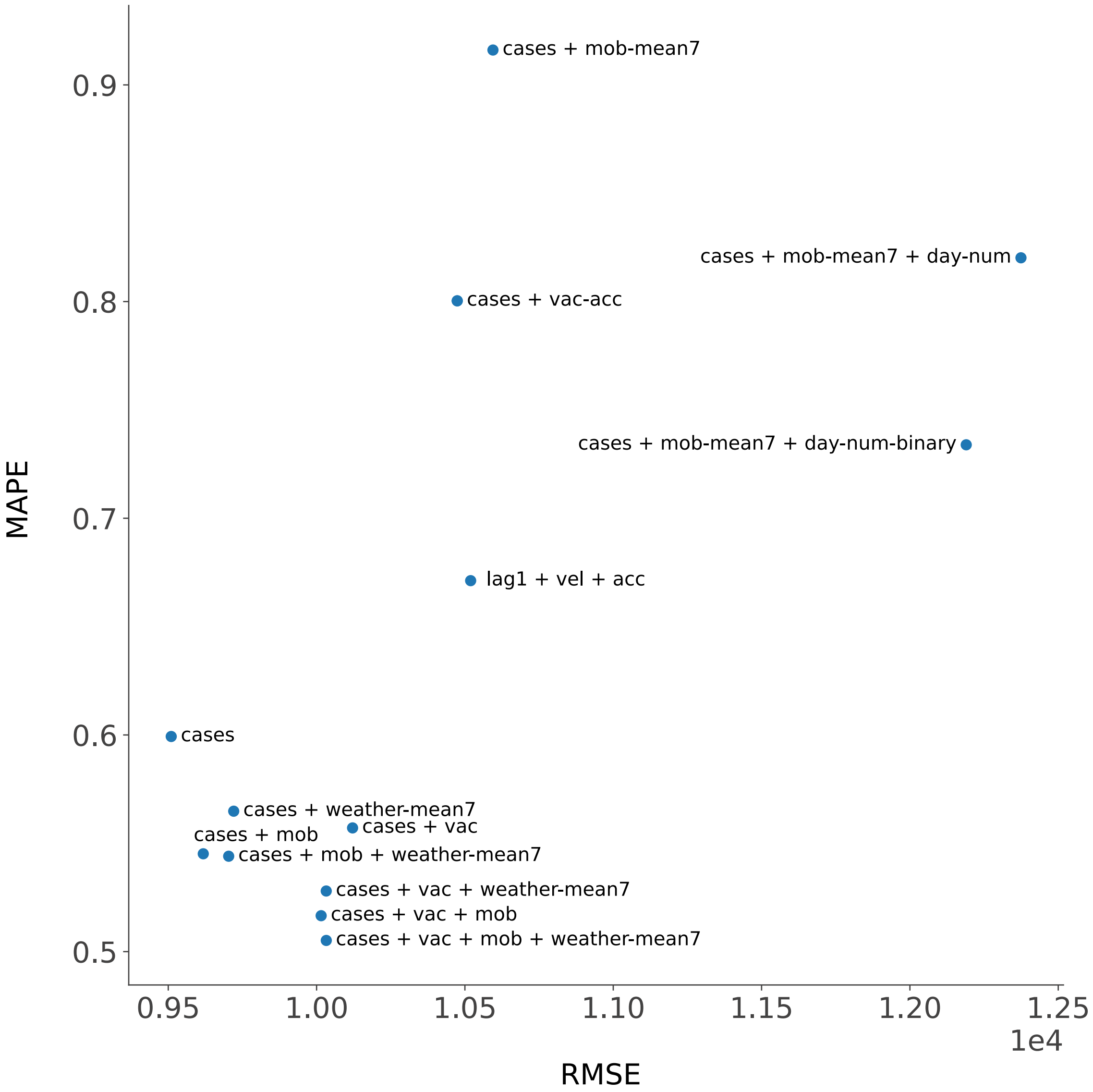

Supplementary figure 2. Performance of some of the additional ML configuration we tried, for the Spain case in the test split. Models are aggregated with mean aggregation.

Test - **full**

		MAPE 1	MAPE 2	MAPE 3	MAPE 4	RMSE 1	RMSE 2	RMSE 3	RMSE 4
**mean**	ML	0.599	0.557	0.517	0.505	9510	10121	10015	10033
	pop	0.521				10006			
	all	0.342	0.350	0.344	0.347	9314	9718	9700	9728
**median**	ML	0.622	0.506	0.461	0.469	9509	10069	9935	9921
	pop	0.501				9537			
	all	0.312	0.335	0.351	0.393	9236	9694	9694	9783
**weighted**	ML	0.583	0.509	0.443	0.422	9507	9858	9603	9624
	pop	0.495				9713			
	all	0.309	0.338	0.336	0.341	9201	9482	9428	9471

Test - **no omicron**

		MAPE 1	MAPE 2	MAPE 3	MAPE 4	RMSE 1	RMSE 2	RMSE 3	RMSE 4
**mean**	ML	1.017	0.762	0.667	0.634	1918	1390	1225	1183
	pop	0.596				1426			
	all	0.280	0.215	0.202	0.207	623	565	569	594
**median**	ML	1.084	0.636	0.520	0.536	2071	1174	1006	1038
	pop	0.594				1423			
	all	0.194	0.177	0.217	0.314	540	536	659	915
**weighted**	ML	0.969	0.648	0.499	0.450	1814	1199	960	914
	pop	0.561				1357			
	all	0.180	0.193	0.198	0.213	498	586	610	663

Test - **omicron**

		MAPE 1	MAPE 2	MAPE 3	MAPE 4	RMSE 1	RMSE 2	RMSE 3	RMSE 4
**mean**	ML	0.324	0.422	0.417	0.420	14517	15880	15813	15869
	pop	0.471				15666			
	all	0.383	0.439	0.438	0.439	15047	15756	15724	15752
**median**	ML	0.318	0.420	0.422	0.424	14414	15937	15825	15780
	pop	0.439				14889			
	all	0.390	0.440	0.440	0.445	14971	15734	15653	15632
**weighted**	ML	0.329	0.417	0.405	0.404	14581	15569	15303	15369
	pop	0.452				15224			
	all	0.394	0.433	0.427	0.426	14941	15349	15244	15281

Supplementary figure 3. MAPE and RMSE obtained in each scenario (`1` , `2` , `3` , `4`) according to each form of aggregation (`mean` , `median` , `weighted`) for different subsets of models (`ML` , `Pop` , `All`). We show averages for three different splits: the full test split, the test split *before* Omicron variant started (mid November), and the test split *after* Omicron variant started. All predictions are made for Spain.

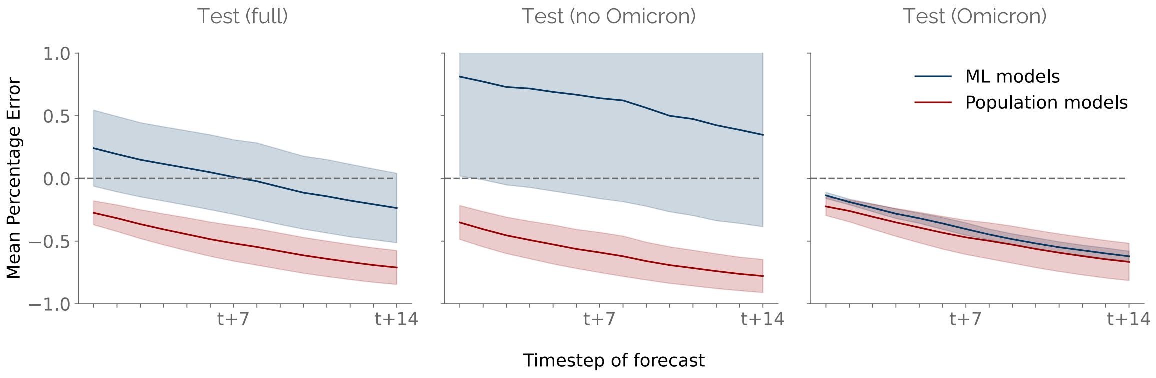

Supplementary figure 4. Mean Percentage Error for each time step of the forecast, grouped by model family. Shades show the standard deviation between models of the same family. ML models are trained in Scenario 4. We show results for three different splits: the full test split, the test split *before* Omicron variant started (mid November), and the test split *after* Omicron variant started. All predictions are made for Spain. The `no-omicron` plot does not exactly reproduce the degradation of ML at large timesteps seen in Figure 9 because the split is not as stable as validation (i.e. Omicron is slowly appearing, driving ML models to underestimation).

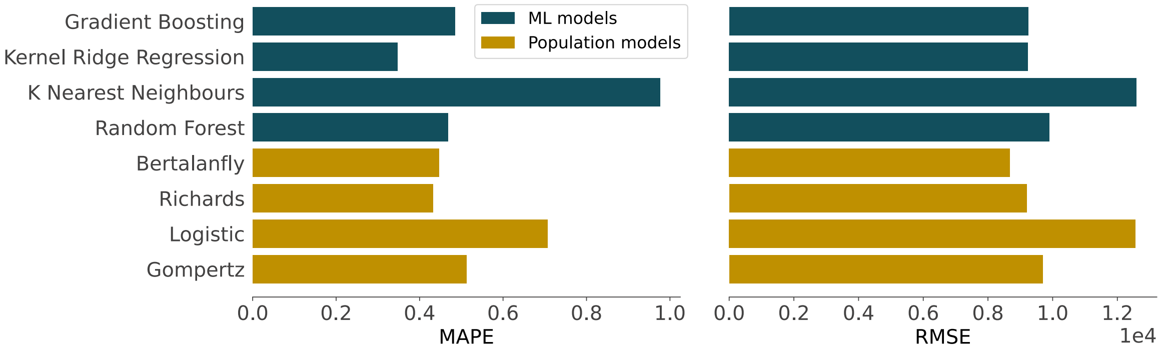

Supplementary figure 5. Mean MAPE and RMSE, for each model, for the Spain case in the test split. ML models are trained in Scenario 4.

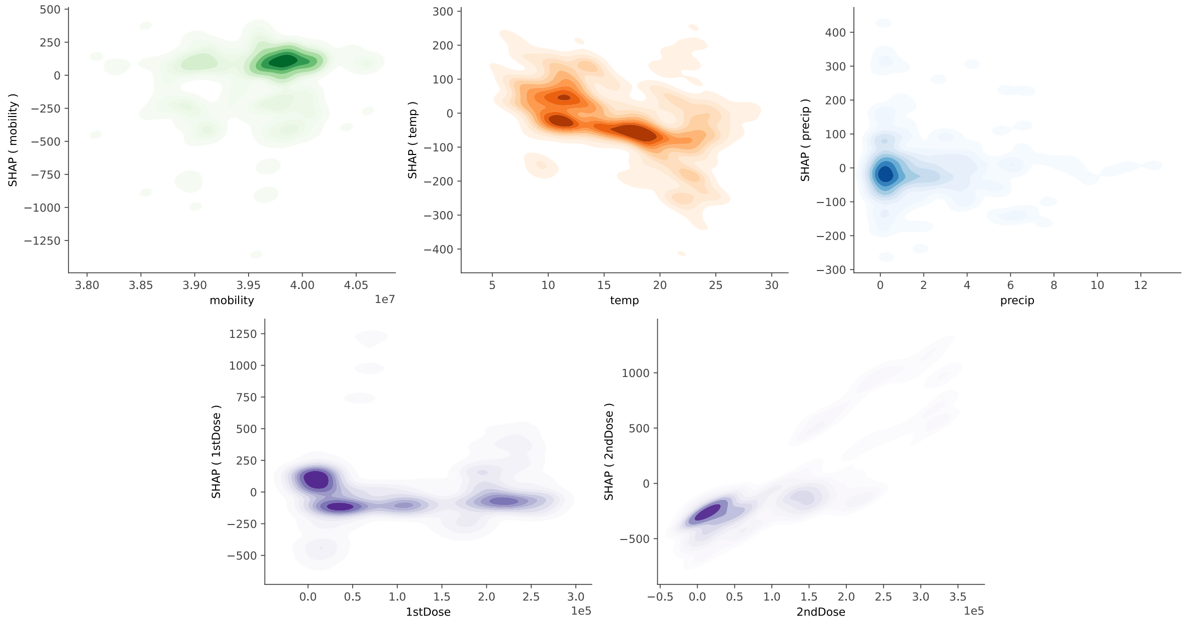

Supplementary figure 6. SHAP dependence plot for non-cases features. For each feature we plot the raw value of the feature vs its associated SHAP value. We average SHAP values across all ML models. All models are trained in Scenario 4 for the Spain case. We display values for all the dataset (train + val + test).

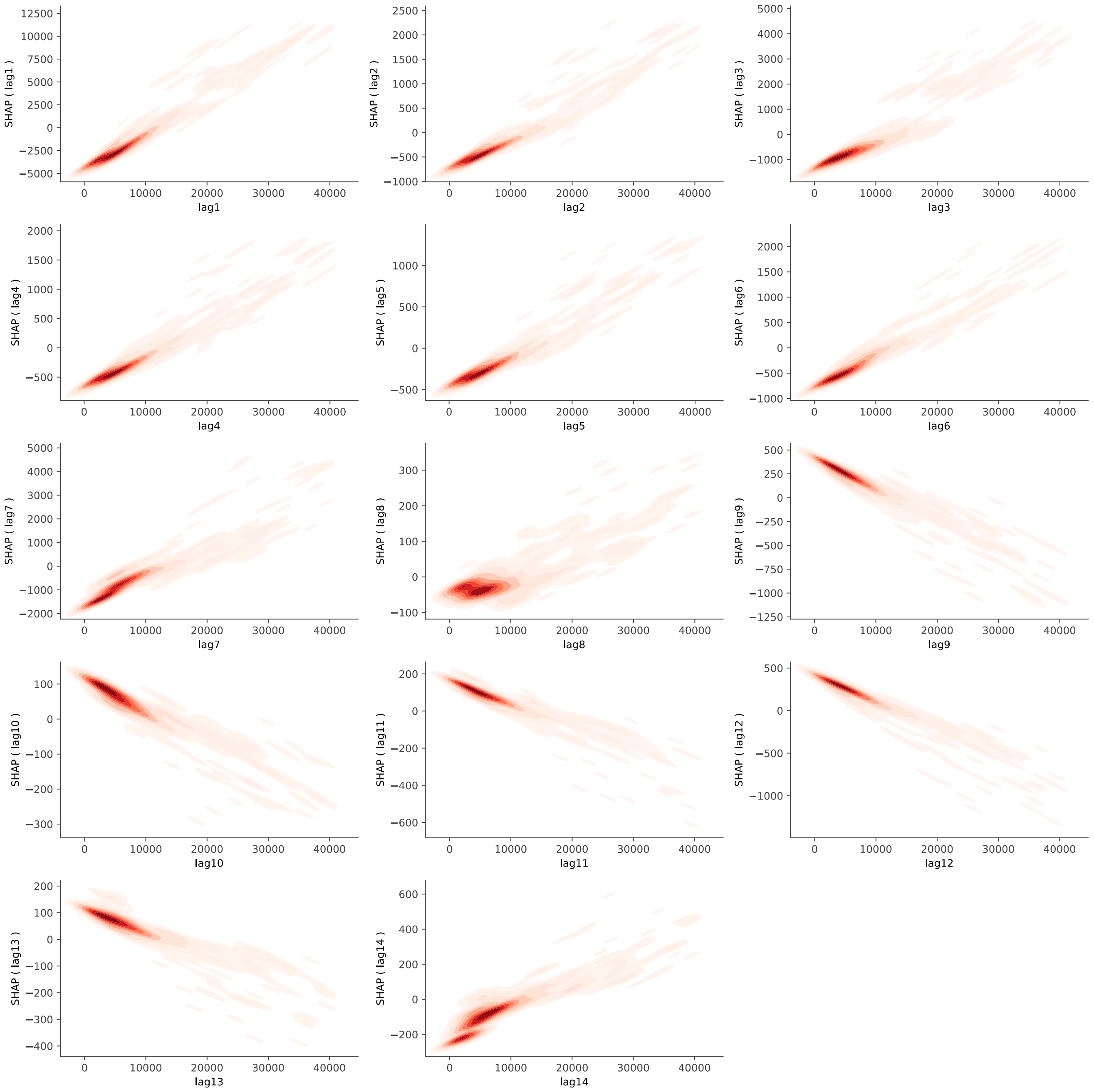

Supplementary figure 7. SHAP dependence plot for cases lags features. For each feature we plot the raw value of the feature vs its associated SHAP value. We average SHAP values across all ML models. All models are trained in Scenario 4 for the Spain case. We display values for all the dataset (train + val + test).

Explicit solution of the ODE of the Gompertz model and estimation of the initial parameters

Remember that the ODE which defines the Gompertz model is given by:

$$\frac{\partial p}{\partial t} = ap(t) - bp(t)log(p(t)), \tag{1}$$

being $p(t)$ the population at time t, and a and b two parameters to determine.

Taking $y(t) = log(p(t))$, we obtain its explicit solution as follows (note that we consider $y'(t) := \frac{\partial y}{\partial t}$ in order to simplify the notation):

$$\frac{\partial p}{\partial t} = ap(t) - bp(t)log(p(t)) \Longrightarrow \frac{\partial y}{\partial t} = a - by(t) \Longrightarrow y'(t) + by(t) = a \Longrightarrow$$
$$\Longrightarrow e^{bt}y'(t) + e^{bt}by(t) = e^{bt}a \Longrightarrow \left(e^{bt}y(t)\right)' = e^{bt}a \Longrightarrow e^{bt}y(t) = a\int e^{bt}dt \Longrightarrow$$
$$\Longrightarrow y(t) = \frac{a}{b} + ce^{-bt} \Longrightarrow \boxed{p(t) = e^{\frac{a}{b}+ce^{-bt}}}$$

In order to estimate the parameters a, b and c we fix three time instants t_i, t_j and t_k verifying: $h = t_j - t_i$ and $2h = t_k - t_i$. Be $\alpha = \dfrac{log(p(t_j)) - log(p(t_i))}{log(p(t_k)) - log(p(t_i))}$ we get:

- $\alpha = \dfrac{e^{-bt_j} - e^{-bt_i}}{e^{-bt_k} - e^{-bt_i}} \Longrightarrow \alpha = \dfrac{1}{1+e^{-bh}} \Longrightarrow b = -\dfrac{1}{h}log\left(\dfrac{1-\alpha}{\alpha}\right)$
- $\dfrac{p(t_j)}{p(t_i)} = e^{c(e^{-bt_j}-e^{-bt_i})} \Longrightarrow c = \dfrac{log(p(t_j)) - log(p(t_i))}{e^{-bt_j} - e^{-bt_i}}$
- $p(t_i) = e^{\frac{a}{b}+c^{-bt_i}} \Longrightarrow a = b\left(log(p(t_i)) - ce^{-bt_i}\right)$

The process to be followed to obtain the initial parameters for the Logistic and Bertalanffy models is analogous to the previous one presented for the Gompertz case.
